# Kanizsa illusory contours appearing in the plasmodium pattern of *Physarum polycephalum*

**DOI:** 10.3389/fcimb.2014.00010

**Published:** 2014-02-28

**Authors:** Iori Tani, Masaki Yamachiyo, Tomohiro Shirakawa, Yukio-Pegio Gunji

**Affiliations:** ^1^Department of Earth and Planetary Science, Graduate School of Science, Kobe UniversityKobe, Japan; ^2^Department of Computer Science, School of Electrical and Computer Engineering, National Defense Academy of JapanYokosuka, Japan; ^3^The Unconventional Computing Centre, University of the West EnglandBristol, UK

**Keywords:** *Physarum*, unconventional computing, Kanizsa illusion, asynchronous automata

## Abstract

The plasmodium of *Physarum polycephalum* is often used in the implementation of non-linear computation to solve optimization problems, and this organismal feature was not used in this analysis to compute perception and/or sensation in humans. In this paper, we focused on the Kanizsa illusion, which is a well-known visual illusion resulting from the differentiation-integration of the visual field, and compared the illusion with the adaptive network in the plasmodium of *P. polycephalum*. We demonstrated that the network pattern mimicking the Kanizsa illusion can be produced by an asynchronous automata-fashioned model of the foraging slime mold and by the real plasmodia of *P. polycephalum*. Because the protoplasm of the plasmodium is transported depending on both local and global computation, it may contain differentiation-integration processes. In this sense, we can extend the idea of perception and computation.

## 1. Introduction

A true slime mold, *Physarum polycephalum* (order Physarales, class Myxomycetes, subclass Myxogastromycetidae) has been used as a model organism to investigate biologically motivated unconventional computing because the plasmodium of *Physarum* can solve various theoretical graph problems, including a Steiner tree, Spanning tree (Adamatzky, 2007), maze (Nakagaki et al., 2000; Nakagaki, 2001), and Voronoi diagram (Shirakawa et al., 2009; Shirakawa and Gunji, 2010). The plasmodium can also perform logical computing (Tsuda et al., 2004; Adamatzky, 2010) and create an optimal adaptive network (Tero et al., 2010; Gunji et al., 2011).

These computations, which produce particular patterns in relation to the input stimulus, were previously distinguished from psychological reactions such as sensation and emotion; however, sensation and emotion are also considered higher-order computations in which pattern formation is applied to the deviation and/or representation of the input stimulus (Jones and Saeed, 2007; Adamatzky et al., 2013; Sakiyama and Gunji, 2014). In this sense, it is possible to say that even an amoeboid animal can feel something (Humphrey, 2006). Whereas the retreat of the marginal front of the amoeba away from a repellant stimulus can be considered a lower-order computation, the reaction to a particular transportation of protoplasm from one margin to the other areas can be regarded as a sensation or a higher-order computation relative to the lower-order one.

When the plasmodium of *Physarum* forages in an environment, the extension or retreat of a local area can lead to the elongation and/or shortening of the tubular network and vice versa. This phenomenon reveals that a lower-order computation can lead to a higher-order computation and vice versa. The synthesis of local and global information propagation can result in a reaction to an external stimulus coupled with a higher-order computation, which might be considered “perception.” This extended perception gives rise to the idea that the plasmodium can emulate and/or compute the perception of a human.

The visual perception of humans is not a simple local reaction to an optical stimulus, such as with a camera. Computing an optical stimulus is not only a local computation but also a global computation, and the local reaction to the optical stimulus can be modified depending on the global propagation of information. A visual illusion is one of the most intriguing examples of visual perception that results from the synthesis of local and global computation. In the Müller–Lyer illusion, the apparent length of a local pattern can be shortened or extended depending on its surrounding pattern (Müller-Lyer, 1896; Howe and Purves, 2005). In the aforementioned sense, biologically motivated computing can reveal visual illusion in terms of the analogy at the phenomenological and mechanistic levels. We previously demonstrated that the distribution of foraging ants attracted to the honey dew along a Müller–Lyer diagram can reveal a Müller–Lyer illusion, wherein the ants in an attractive field can be compared to the neurons in a visual field (Sakiyama and Gunji, 2014). Not only ants but also the plasmodium of *Physarum* can emulate various visual illusions because the plasmodium integrates lower- and higher-order computations. In this study, we focus on a particular visual illusion, Kanizsa illusory contours.

Kanizsa illusory contours are subjective shapes induced from local pac-man elements (Kanizsa, 1979). According to Gestalt psychology, human vision is sensitive not to the input stimulus *per se* but to the relative values of input signals (Koffka, 1935). This idea, when theoretically enforced (Ross and Pessoa, 2000), explains that the Kanizsa illusion is triggered by the relative contrast in the Kanizsa diagram, developed by contour completion and finally completed by filling in (Pessoa et al., 1998).

Although the process of contour completion was first explained using a simple collinearity-based model (Grossberg, 1994; Grossberg and Pessoa, 1998), the disappearance of illusion cannot be explained by this model when the pac-man elements in the Kanizsa figure are replaced by crosses. The simple filling-in is unable to explain the perceived brightness and/or occlusion of the central area of the Kanizsa illusory contours, as noted by Coren (1972). To resolve these problems, a differentiation-integration approach is employed by some models (Gillam and Nakayama, 2002). The edge of the bright area is detected by differentiation, and the contrast of the pac-man is also preserved in a memory. Through integration, this information creates a line connecting the pac-men, the brighter surface in the central area and the relative depth featuring the occlusion cues (Kogo et al., 2010). By introducing a particular mechanism in which the local elongations of contours interact and are canceled, the disappearance of the illusion for a particular variation of a Kanizsa figure can be explained.

When the plasmodium of the *Physarum* is considered, the Kanizsa illusory contours might be emulated by a real foraging plasmodium. We previously proposed an asynchronously updated automata-fashioned model for the plasmodium of the *Physarum* (Gunji et al., 2008b,a, 2011; Niizato et al., 2010). This model can mimic amoebic motions, adaptive networks and growth patterns in unstimulated environments of the plasmodium of *Physarum*. Because this model is based on the local detection of the environmental stimulus and the asynchronous transportation of protoplasm, the model can embed the synthesis of the differentiation-integration process. In this sense, the model and/or the real plasmodium of *Physarum* can connect separated pac-men by a tubular structure, which reveals an illusory contour. We ignore the brighter surface and occlusion cues of the central illusory area in the Kanizsa illusion and focus only on the contour creation. Because the filling-in and the relative depth can be simulated if our *Physarum* model is coupled with a particular mechanism, the significance of our model and the real *Physarum* should not be lost.

In this paper, we demonstrate that our model for foraging *Physarum* can reveal Kanizsa illusory contours if the pac-man faces the center, whereas the illusory contours disappear if the pac-man faces outward. In this result regarding stimulation, the pac-man is implemented as an attractant in the automata-fashioned model. We also demonstrate that the same patterns are obtained in the experiment involving the real plasmodium of the *Physarum*. In this case, the pac-man is implemented using a nutrient-rich dry agar sheet. These observations suggest that the transfer of biological information resulting from the synthesis of local and global interactions can create what is called extended visual perception, thus allowing visual illusion, i.e., biological and/or unconventional computing can emulate the perception of humans.

## 2. Materials and methods

### 2.1. Definition of the model

Models of *Physarum* plasmodium have been suggested. For example, weighted graph model (Tero et al., 2006, 2007), particles model (Jones, 2010), and network growth model (Takamatsu et al., 2009), and so on. We use the Vacant-Particle (VP) model proposed by Gunji et al. (2008a,b) to explain the adaptive network of the plasmodium of *Physarum*. This is an asynchronous cellular automaton defined on a discrete lattice plane in which the asynchronous application of transition rules provides amoeboid motility. This model has the advantage of not only simulating the amoeboid cellular motion of *Physarum* but also forming adaptive networks that depend on the surrounding environment (Niizato et al., 2010; Gunji et al., 2011; Tani et al., 2011).

**Figure 1:**
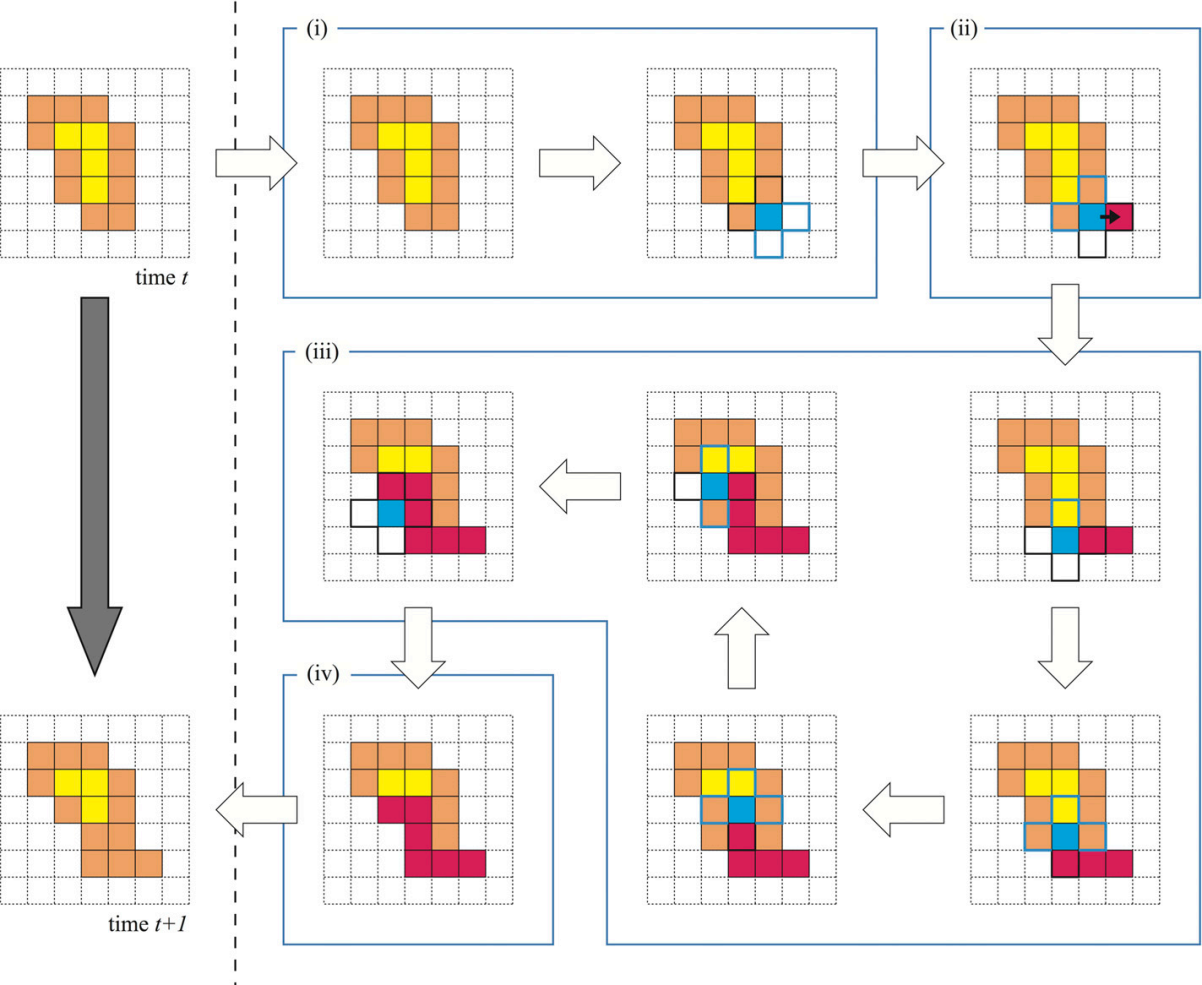
**Basic algorithm of the model**. The left column indicates the time evolution of the model from time *t* to *t* + 1. The right-hand portion indicates the four child processes: (i) the choice of stimulus point; (ii) the growth to the outside and entry into the VP; (iii) the migration of VP with a gel transition; and (iv) the escape of VP.

First, we explain the basic algorithm of the model. The additional rules coupled with the model are defined later. The coordinates of a site on the discrete lattice plane are described by a pair of integers (*i,j*). For all sites (*i,j*), a binary value *c*(*i,j*) is assigned. *c*(*i,j*) = 1 indicates that the site (*i,j*) is filled with the plasmodium and implies that the site is inside of the plasmodium. These are called internal sites. Similarly, *c*(*i,j*) = 0 indicates that the site (*i,j*) is outside the plasmodium. These are called external sites. In particular, an internal site that confronts at least one external site in a von Neumann neighborhood is defined as a membrane. In the left column of Figure 1 the normal internal sites are yellow, and the membrane sites are orange, whereas the external sites are not colored.

We use the symbol *N* to indicate an aggregation of internal sites.

(1) N={(i,j) | c​(i,j)=1}

Similarly, the symbol *M* indicates an aggregation of membrane sites.

(2) $$\begin{array}{l} M=\text{​}\{\left(i,j\right)\in N|c\text{​}\left(i+1,j\right)\text{​}=\text{​}0\vee c\text{​}\left(i-1,j\right)=0\vee c\text{​}\left(i,j+1\right)\\ \;=0\vee \text{​}c\text{​}\left(i,j-1\right)\text{​}=0\} \end{array}$$

Next, we explain the processes that provide amoeboid cell motility for *N*. Note that the word “neighborhood” means “von Neumann neighborhood.”

The temporal transition of a plasmodium from time *t* to time *t* + 1 is illustrated in the left column of Figure 1. There are four child processes in the background of this change, and these are depicted in the right part of Figure 1(i–iv).

- We randomly choose one site from *M*. This selected site is called the stimulus point (SP). The SP is colored blue in Figure 1(i).
- Because the SP is a membrane cell, there is at least one external site in the neighborhood of the SP. We also randomly select an external site from among these neighbors. This selected external site is called the growth point (GP). The protoplasm at SP then moves to GP. This movement means that the value *c* at the GP changes to 1. Instead of this movement, an external void inserts into the SP. The void that has entered the plasmodium is called the VP.
- The VP migrates between the internal sites *N* by exchanging places with neighboring internal sites. This sequential movement of the VP mimics a protoplasmic flow. The most important condition is that the protoplasm experiencing an exchange itself produces a gel transition. Because of this, these cells are never exchanged with the VP again. Therefore, the migration pathway of the VP never intersects with itself. The red-colored sites in Figure 1(iii) indicate the gelled protoplasm. The VP continues searching exchangeable cells in the neighborhood and exchanging places if possible.
- In the case where the VP has no exchangeable neighborhood sites or the VP has exchanged a particular given number of times, the VP escapes from the plasmodium. This site is called the escape point (EP). Then, the value *c* at the EP becomes 0. Eventually, we observe that the protoplasm that was at the EP has moved to the GP. Therefore, these processes conserve the number of internal sites. i.e., the covered area by plasmodium does not change temporally. Finally, the entire gelled protoplasm undergoes a sol transition.

This is the basic algorithm of the model. These processes are called the “one time step,” i.e., they occur all at once. The characteristic of this model is a gel transition. This condition maintains the integrity of the plasmodium and enables amoeboid motion. However, this basic model *Physarum* cannot move over a long distance because the random selection of the SPs and GPs prohibits unidirectional growth. Hence, to acquire real *Physarum* plasmodium-like behavior, some taxis must be defined regarding this model. In fact, the introduction of the localization of probabilities to the random choice process yields taxis for the model.

### 2.2. Additional rules

It is well known that real *Physarum* plasmodium has a negative taxis to white light. Thus, we propose an Avoid Bright region VP (ABVP) model as defined below.

For all sites (*i,j*) on the lattice plane, a new binary value *b*(*i,j*) is defined. This value *b*(*i,j*) corresponds to the brightness of the site. *b*(*i,j*) = 1 indicates that the site is bright, and *b*(*i,j*) = 0 indicates that the site is dark.

In the basic model, there are three types of random choice processes, which are detailed below.

1. The choice of the SP.
2. The choice of the GP.
3. The choice of the direction of movement of the VP.

For each of these processes, we introduce variations of probabilities.

#### 2.2.1. The choice of the SP

**Figure 2:**
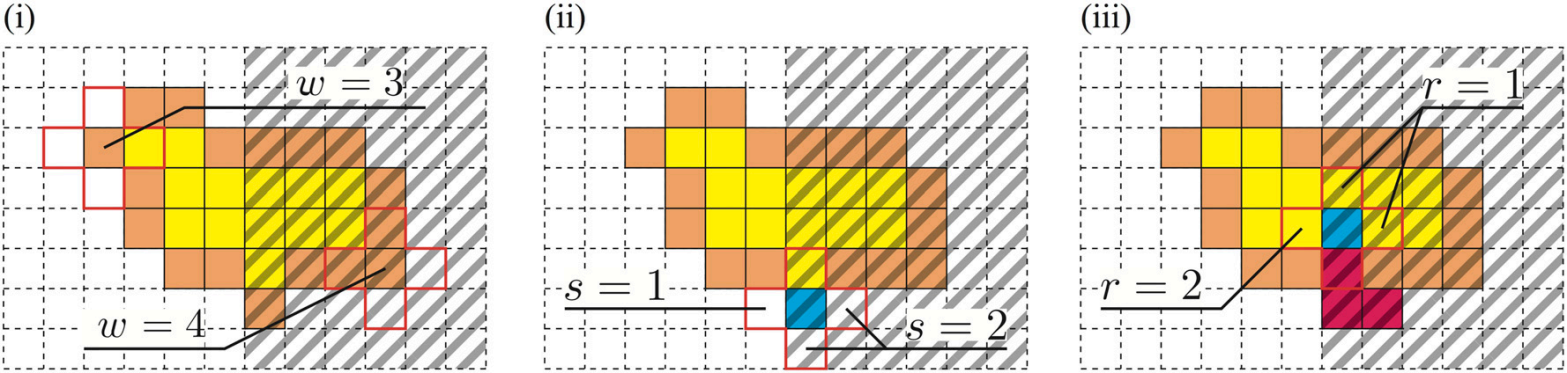
**The right part of each figure is dark, and the other area is bright**. (i) The membrane at the dark site is chosen more frequently. (ii) Growth is attracted to the dark site. (iii) Avoidance of the bright area.

First, for all sites (*i,j*), we define the integer value *s*(*i,j*) depending on *c*(*i,j*) and *b*(*i,j*). The value *s*(*i,j*) is determined as follows [Figure 2(i)]:

(3) $$s(i,j)=\{\begin{array}{rr} 0 & \left(c\text{​}\left(i,j\right)=1\right)\\ 1 & \left(c\text{​}\left(i,j\right)=0\wedge b\text{​}\left(i,j\right)=1\right)\\ 2 & \left(c\text{​}\left(i,j\right)=0\wedge b\text{​}\left(i,j\right)=0\right). \end{array}$$

Next, the new value *w*(*i,j*) is defined for all of the membrane sites depending on *s*(*i,j*), as defined in the equation below.

(4) w​(i,j)=s​(i+1,j)+s​(i−1,j)+s​(i,j+1)+s​(i,j−1). 

Here, *w*(*i,j*) reflects the local environment of the site (*i,j*). This *w*(*i,j*) value determines the probability *p*_*s*_(*i,j*) that the membrane site (*i,j*) will be selected as the SP.

(5) $$p_{s}\text{​}\left(i,j\right)=\frac{w\text{​}\left(i,j\right)}{\sum_{\left(m,n\right)\in M}w\text{​}\left(m,n\right)}.$$

Therefore, the membrane sites that have many external and dark neighborhoods are selected with a high probability.

#### 2.2.2. The choice of the GP

Let a membrane site (*i,j*) be selected as the SP. The GP is then determined using the probabilities described below.

For a neighbor site of the SP (*i,j*), which for convenience we write as (*i*′,*j*′), the probability *p*_*g*_(*i*′,*j*′) that the site (*i*′,*j*′) will be selected as the GP is determined by the value *s* that was previously defined in [Figure 2(ii)].

(6) $$p_{g}\text{​}\left(i^{\prime },j^{\prime }\right)=\frac{s\text{​}\left(i^{\prime },j^{\prime }\right)}{s\text{​}\left(i+1,j\right)+s\text{​}\left(i-1,j\right)+s\text{​}\left(i,j+1\right)+s\text{​}\left(i,j-1\right)}.$$

Note that if a neighboring site is internal, then the probability choice becomes 0 because of the definition of *s*.

#### 2.2.3. The choice of the direction of movement of the VP

The above two conditions promote biased growth toward the dark sites. Next, we introduce another rule to implement repellency from the bright region. To decrease the plasmodium under the bright area, the VP is expected to move toward the brighter area. Let the VP be at the site (*i, j*); then for all neighbor sites (*i*′,*j*′) of the VP, a value *r*(*i*′,*j*′) is defined as follows [Figure 2(iii)]:

(7) $$r(i^{\prime },j^{\prime })=\{\begin{array}{rr} 0 & \left(c\text{​}\left(i^{\prime },j^{\prime }\right)=0\vee \left(i^{\prime },j^{\prime }\right)\text{is gelled}\right)\\ 1 & \left(c\text{​}\left(i^{\prime },j^{\prime }\right)=1\wedge \left(i^{\prime },j^{\prime }\right)\text{is not gelled}\wedge b\text{​}\left(i^{\prime },j^{\prime }\right)=0\right).\\ 2 & \left(c\text{​}\left(i^{\prime },j^{\prime }\right)=1\wedge \left(i^{\prime },j^{\prime }\right)\text{is not gelled}\wedge b\text{​}\left(i^{\prime },j^{\prime }\right)=1\right). \end{array}$$

The probability *p*_*m*_(*i*′,*j*′) that the neighbor site (*i*′,*j*′) will be chosen as the next destination of the VP is then determined as in the following equation:

(8) $$p_{m}\text{​}\left(i^{\prime },j^{\prime }\right)\text{​}=\text{​}\frac{r\text{​}\left(i^{\prime },j^{\prime }\right)}{r\text{​}\left(i+1,j\right)+r\text{​}\left(i-1,j\right)+r\text{​}\left(i,j+1\right)+r\text{​}\left(i,j-1\right)}.$$

Note that if the value *r*(*i*′, *j*′) = 0 for all neighborhood (*i*′, *j*′) values, i.e., the probabilities *p*_*m*_(*i*′, *j*′) = 0 for the entire neighborhood, then the escape process occurs according to the basic algorithm.

However, the effects of this condition are very restrictive in the sense that only local information of the VP is used, and this correction is valid only when the VP is at the edge of a bright/dark region.

#### 2.2.4. Linear structure correction

An internal site (*i, j*) is called a “linear structure” if both sides of (*i, j*), (*i* + 1, *j*), (*i* − 1, *j*) or (*i, j* + 1), (*i, j* − 1) are external sites.

During the process of randomly choosing a stimulus point, if a candidate site (*i,j*) is a linear structure, then a positive constant *L* is added to *w*(*i,j*). From previous work (Gunji et al., 2011), it is known that a larger value of *L* produces a straighter linear network. However, a value for *L* that is too large will invalidate the negative taxis for the bright regions. Therefore, in this paper, the value of *L* was set to 10.

#### 2.2.5. Thickness rules

In the basic algorithm, the number of internal sites does not change throughout the development. This implies that the area covered by the model plasmodium is unchanged. A real *Physarum* plasmodium, however, changes its cell thickness spatiotemporally to spread widely or concentrate in a narrow area. In particular, under the condition that we consider here, a real plasmodium would likely converge upon dark regions, and its cell thickness would increase.

To implement this behavior, we add the next two rules to our model. Let (*i*_*g*_, *j*_*g*_) be the GP and (*i*_*e*_, *j*_*e*_) be the EP at the time step *t*.

After the escape process,

1. if *b*(*i*_*g*_, *j*_*g*_) = 0 and *b*(*i*_*e*_, *j*_*e*_) = 1, then *c*(*i*_*g*_, *j*_*g*_) = 0,
2. if *b*(*i*_*g*_, *j*_*g*_) = 1 and *b*(*i*_*e*_, *j*_*e*_) = 0, then *c*(*i*_*e*_, *j*_*e*_) = 1.

**Figure 3:**
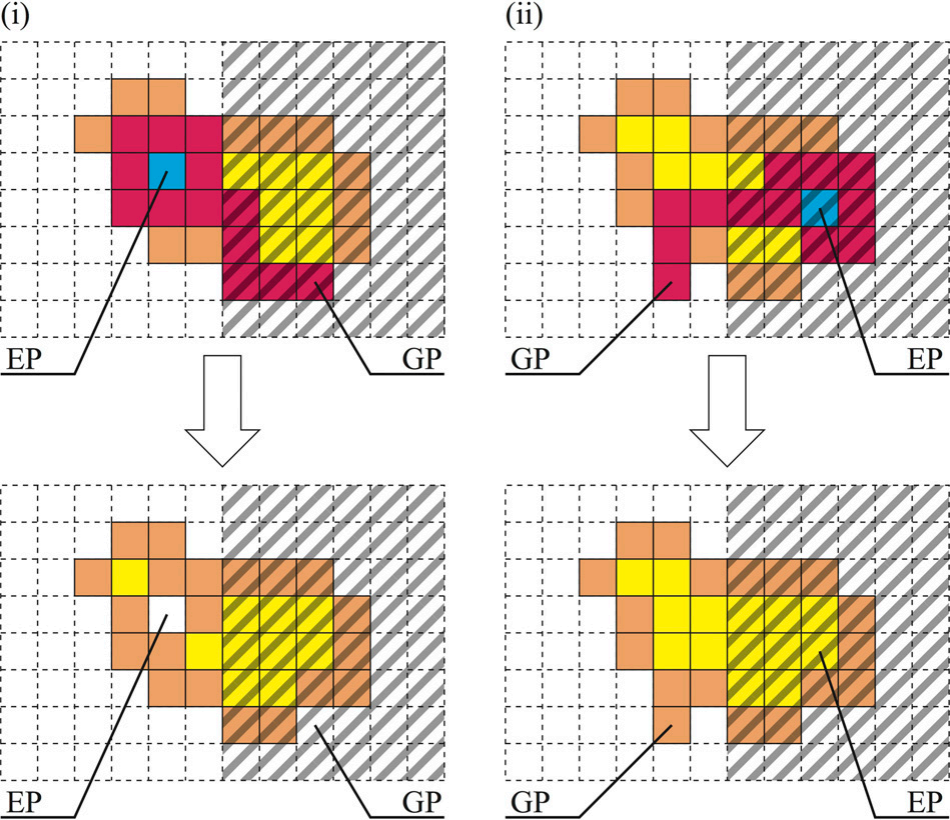
**Thickness rules**. (i) If GP is dark and EP is bright, then *c*(GP) = 0, and the covered area decreases. Conversely, (ii) if GP is bright and EP is dark, then *c*(EP) = 1, and the covered area increases.

What do these rules mean? (1) If the protoplasmic flow moves from a bright region to a dark region, then protoplasm is used to increase the thickness at the SP. Accordingly, the growth process appears to be halted [Figure 3(i)]. (2) In contrast, if the protoplasm moves from a dark region to a bright region, then the cell thickness at the dark region decreases instead of protoplasmic spreading at the bright region. This rule invalidates the escape process [Figure 3(ii)].

The former rule decreases the area covered by the plasmodium, and the latter rule increases this area. These rules are applied deterministically depending on only the brightness at the GP and the EP.

#### 2.2.6. Fluctuation

The final additional rule is related to fluctuations. At each time step, the definition of the value *s*, which was given in section 2.2.1, is changed with the probability *p*_*f*_ such that

(9) $$s\text{​}\left(i,j\right)=\{\begin{array}{ccc} \begin{array}{c} 0\\ 1 \end{array} & \begin{array}{c} \left(c\text{​}\left(i,j\right)=1\right)\\ \left(c\text{​}\left(i,j\right)=0\right) \end{array} & \left(\text{with }p_{f}\right). \end{array}$$

The linear structure correction is not applied here. Therefore, the stimulus point is chosen depending on only the local information regarding the state of the sites. Because the SP is chosen uniformly, this rule accordingly promotes growth in the bright region, which results in the reconstruction of the networks. As a result, shorter networks are given. In this paper, we set *p*_*f*_ = 0.05.

### 2.3. Experimental configuration

#### 2.3.1. The model

**Figure 4:**
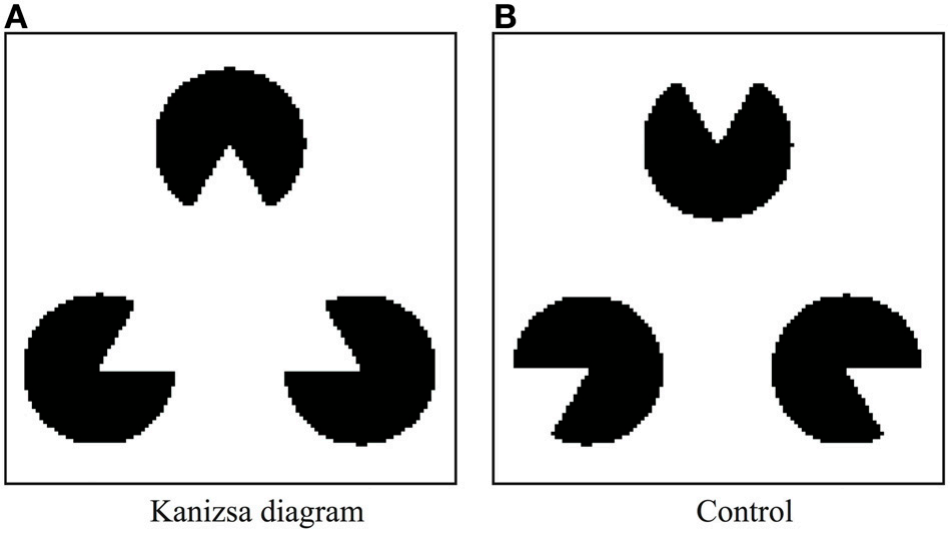
**Stimulus figures are given as geometrical patterns of brightness (black or white)**. **(A)** Kanizsa diagram and **(B)** control figure.

A detailed description of our *Physarum* model is provided herein. Pac-men in the Kanizsa diagram are defined as dark regions, and other areas are defined as bright regions (Figure 4A). For the control experiment, the pac-men in the Kanizsa diagram are turned 180° (Figure 4B). Clearly, in the case of the control, the illusory contour does not appear in the perception of humans. The resolution of both of these images is 120 × 120 pixels. The lattice plane on which the models are defined has the same size.

We conducted experiments 100 times for each image and examined the network topology obtained after five million time steps for each experiment. The maximum limit for the number of VP movements for each time step was set at 300. The initial state of the plasmodium was determined using the following method:

1. For all sites on the lattice plane, we defined *c* = 1;
2. A site that has a value *c* = 1 was randomly chosen as the SP;
3. The VP enters the plasmodium at SP, although growth does not occur;
4. The VP migrates between the internal sites conforming to the rules of the VP model and eventually stops;
5. The site at which the VP stops becomes *c* = 0 (Because the growth process was halted, the area covered by the plasmodium decreases by one site);
6. Repeat 2–5 until the entire covered area is reduced to <45% of the area of the entire lattice plane.

This method provides cancellate networks that cover the entire plane with many medium-size holes. These networks imitate the real *Physarum* tubular networks that are formed on an unstimulated agar plate.

In addition to simulation studies, we conducted a real plasmodium experiment in which pac-men in the Kanizsa diagram are implemented with nutrient rich agar (attractant). In the next section, we describe the experimental configuration for a real plasmodia experiment.

#### 2.3.2. Real plasmodia

We cultivated *Physarum* plasmodia using the method of Camp (1936). Briefly, the plasmodia were cultured on wet paper towels laid out on glass Petri dishes in a plastic box. The space below the towels was filled with tap water to maintain humidity. Oatmeal flakes were provided daily as food. We maintained the culture environment in the dark at 25°C.

In this study, we introduced a sheeted food source made from nutrient-rich agar to make attractant figures. The sheet was easily shaped into various figures and sizes. The nutrient-rich sheet was produced as follows: 400 ml of a solution containing 1.5% agar and 20 mg/ml oatmeal powder was poured into a 295 × 220 mm plastic tub; the resulting sheet was dried thoroughly in a natural convection oven at 50°C.

**Figure 5:**
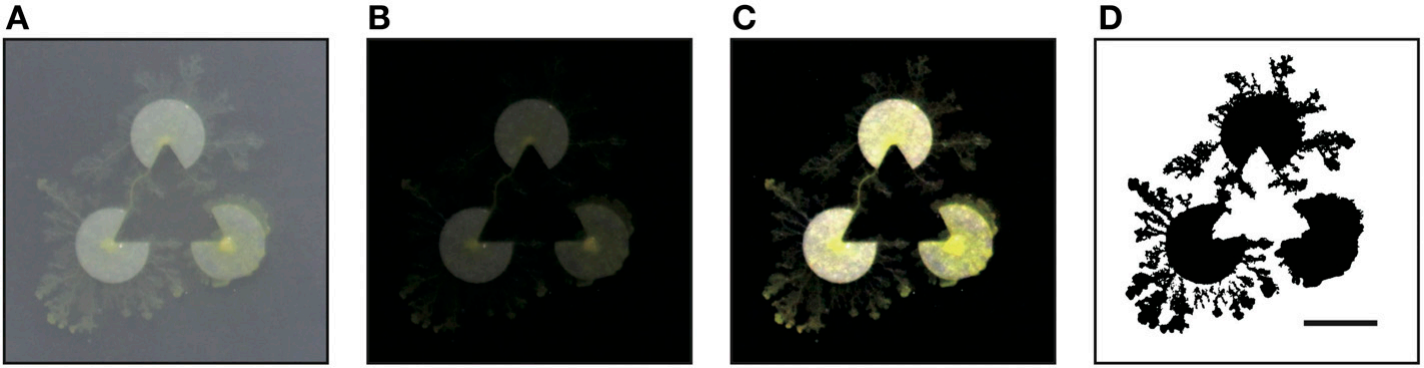
**Experiment using real *Physarum* (scale bar: 10 mm)**. The radius of a pac-man was 5 mm, and the distance between the centers of two pac-men was 15 mm. **(A)** The captured image. **(B)** Obtained by subtracting the background of **(A)**. **(C)** Adjusted the brightness. **(D)** Binarization. All processes were executed by using the ImageJ software. In the binary image, the plasmodia are indicated by black pixels. The threshold of binarization is determined not to delete delicate structure of plasmodium.

In the experiment, we made three pac-men from the above nutrient agar sheet. The radius of a pac-man was 5 mm, and the distance between the centers of two pac-men was 15 mm (Figure 5). We used a 1.5% unamended agar plate in a 9 cm diameter Petri dish as an experimental field. We put the agar sheet pac-men onto the plain agar plate in a fixed arrangement and kept it at rest until the sheets were rehydrated (approximately 1 h). Next, we inoculated 5 mg of plasmodial fragments onto the center of each of pac-man and photographed the plasmodia at intervals of 30 min. We captured images using a single-lens digital camera (EOS Kiss X6, Canon, Japan) from directly above, and the experimental system was kept at 25°C. We repeated the experiments 24 times and performed the same number of control experiments.

## 3. Results

### 3.1. Model results

**Figure 6:**
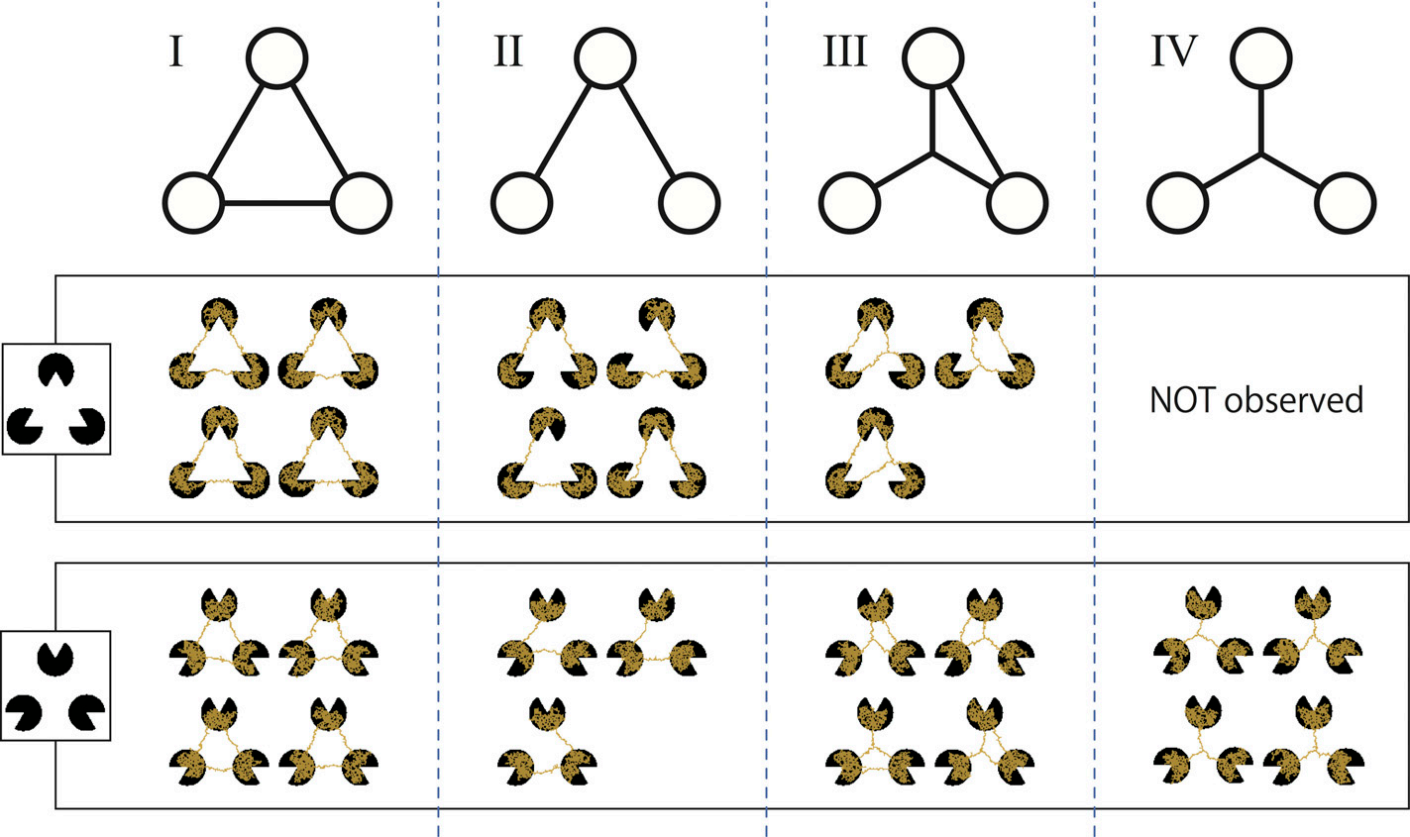
**Classification of the resulting networks according to their topology**. Type I is a triangle; type II is a V-shape; type III is a slingshot-like shape; and type IV is a star or Y-shape. A type IV network was not obtained for the Kanizsa diagram.

We classified the resulting networks based on their topology. The networks were divided into four types (Shirakawa and Gunji, 2007) (Figure 6) as follows:

- Type I is a triangle that connects three pac-men;
- Type II is a V-shape obtained from a triangle lacking of side;
- Type III has another node that links with all of the pac-men and one side of a triangle;
- Type IV is a simple star structure.

However, for the Kanizsa diagram, there were a few undifferentiated networks that could not be classified any of the above types. Therefore, all of networks these were categorized together as “others” and excluded from the following statistical test. Obviously, type I networks complement the illusory contour of a Kanizsa diagram.

The frequencies of each type are given in Table 1. Clearly, in the case of the Kanizsa diagram, type I networks were obtained more frequently than the control case. Type IV networks were not obtained at all, while this type comprised over 40% of the resulting networks in the control case. A χ-square test indicated that there was a significant difference between these two data [χ-square(3, *N* = 191) = 56.7, *p* < 0.001]. Therefore, this difference in the frequencies between the Kanizsa triangle and the control figure reflects the difference in the configuration of the pac-men.

**Table 1:** **The frequency of network types for two stimulus patterns**.

**Stimulus image**	**Type I**	**Type II**	**Type III**	**Type IV**	**Others**	**Total**
Kanizsa diagram	79	9	3	0	9	100
control	46	3	8	43	0	100

**Figure 7:**
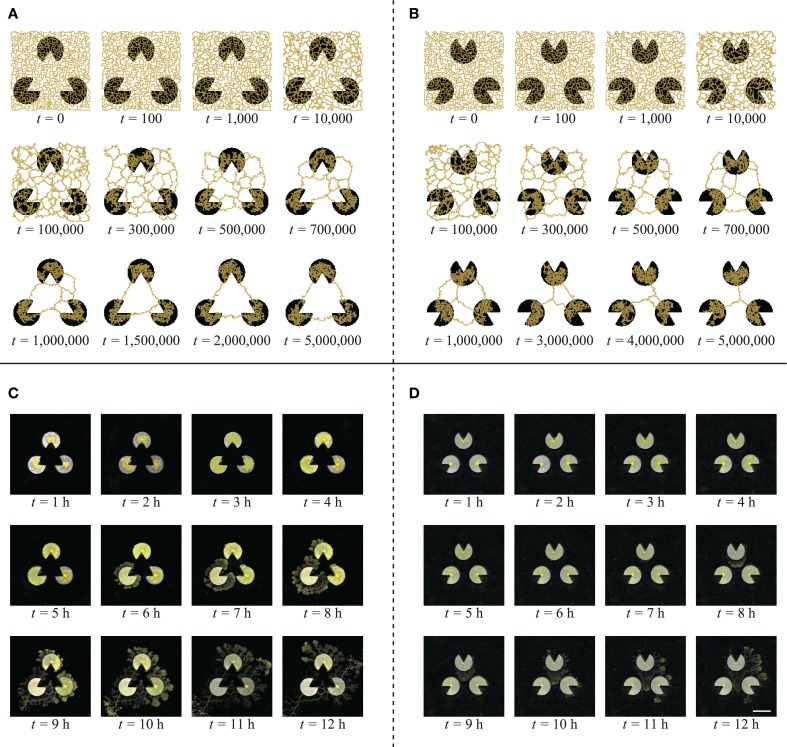
**Typical evolution of the networks of model (A,B) and real plasmodium (C,D) (scale bar: 10 mm)**. **(A)** For the Kanizsa diagram, the network structures are highly stable. **(B)** In the control diagram, the network changes gradually and becomes shorter. **(C)** The plasmodium does not enter the central area of the illusory triangle. **(D)** The plasmodia occupy the middle area of the pac-men.

What was the cause of this difference? To clarify this, we illustrate an example of the time development of the network for each stimulus image (Figures 7A,B). In the case of the Kanizsa diagram, the illusory contour is very stable. It formed completely before *t* = 2,000,000 and was maintained until *t* = 5,000,000. In contrast, in the control, the network changed over time and was not stable. This is a general disposition of the types in Table 1. According to this perspective, the explanation of the difference is a difference in the stability of the *Physarum* network for each stimulus image. This suggests the presence of a unique potential structure for each image. The lack of a mouth for the pac-man may work as some sort of “energy barrier” for the plasmodial networks. In the Kanizsa diagram, these barriers produced the triangular networks because all of the pac-men were arranged face-to-face. However, the configuration of the control figure did not work similarly. In this sense, the resulting networks reflected the global configuration of the pac-men.

### 3.2. Results using real plasmodia

**Figure 8:**
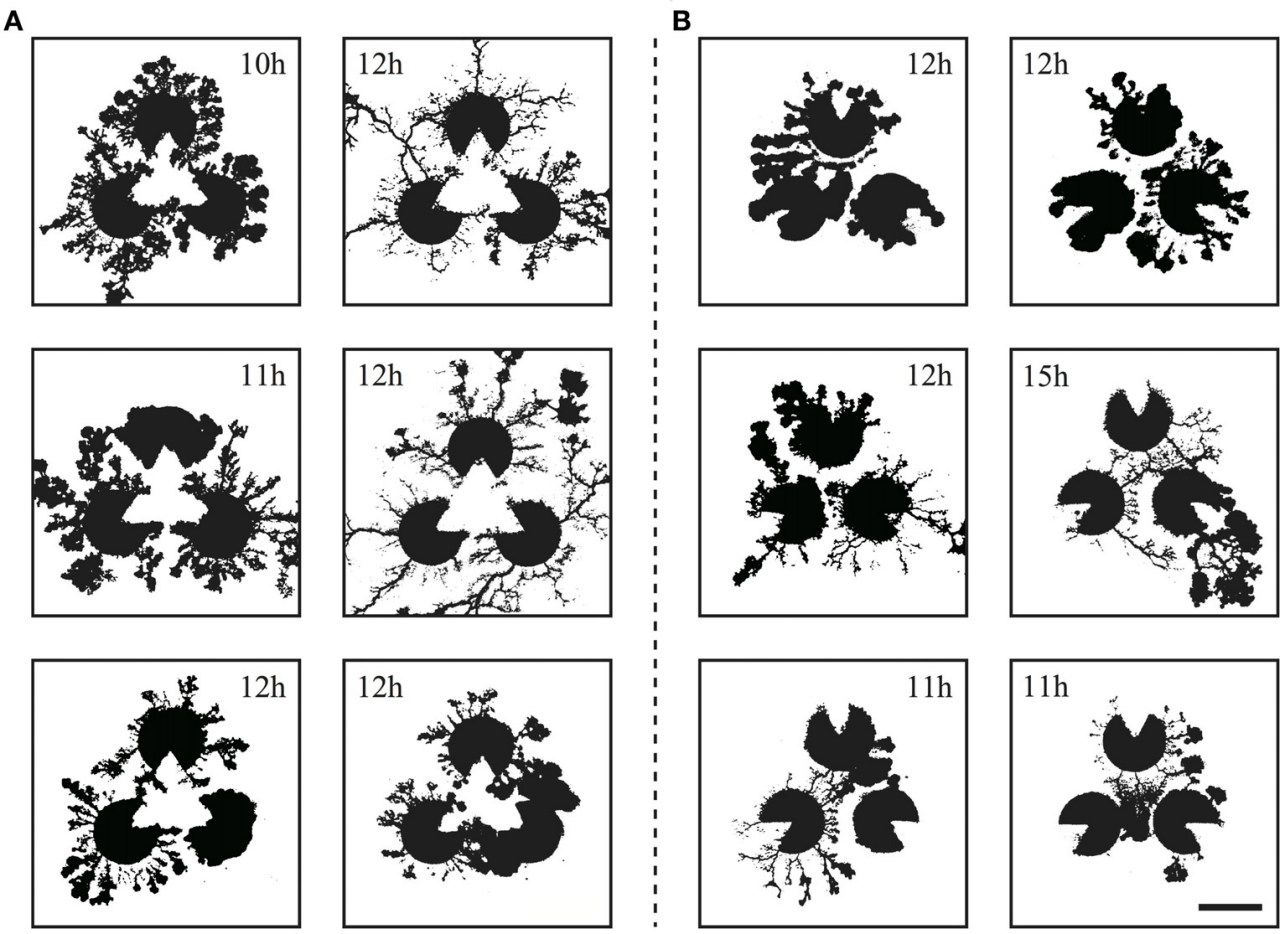
**The resulting networks from the real *Physarum* experiment (scale bar: 10 mm) corresponding to the Kanizsa diagram **(A)** and the control **(B)****. The time stamp of each networks are shown in the image. Note that these networks are the results of individual trials and these photos are not in a time series.

In these experiments, we compared real *Physarum* networks with our model. Because the real plasmodia networks usually have more complex structures than our model (Figure 8), the classification of network types is not obvious, unlike the model cases. Therefore, we adopted another analytical method to estimate the spatial distribution of the plasmodia.

The captured images of the real plasmodia were processed using ImageJ image processing software (Rasband and ImageJ, 1997–2012). Figure 5 shows each step of the binarization process for the image. Figure 5A is the original photograph, and **(B)** is obtained using “Subtract Background”, a macro function of ImageJ, on **(A)**. The “Adjust/Brightness and Contrast” command to automatically normalize the brightness produces image **(C)**. The threshold of binarization is determined according to the brightness value of the site on which the plasmodia are thinly spread to maintain the delicate structures. Obvious noise surrounding the plasmodium, such as dust or bubbles in the agar plate are removed manually. Finally, the binarized image **(D)** is obtained. The time series of plasmodial networks are shown in Figures 7C,D, and some examples of the binarized images of the networks are shown in Figure 8. According to these images, the plasmodium rarely entered the central area of the illusory triangle in the case of the Kanizsa diagram. In contrast, the corresponding areas of the control figures are occupied by the plasmodium.

**Figure 9:**
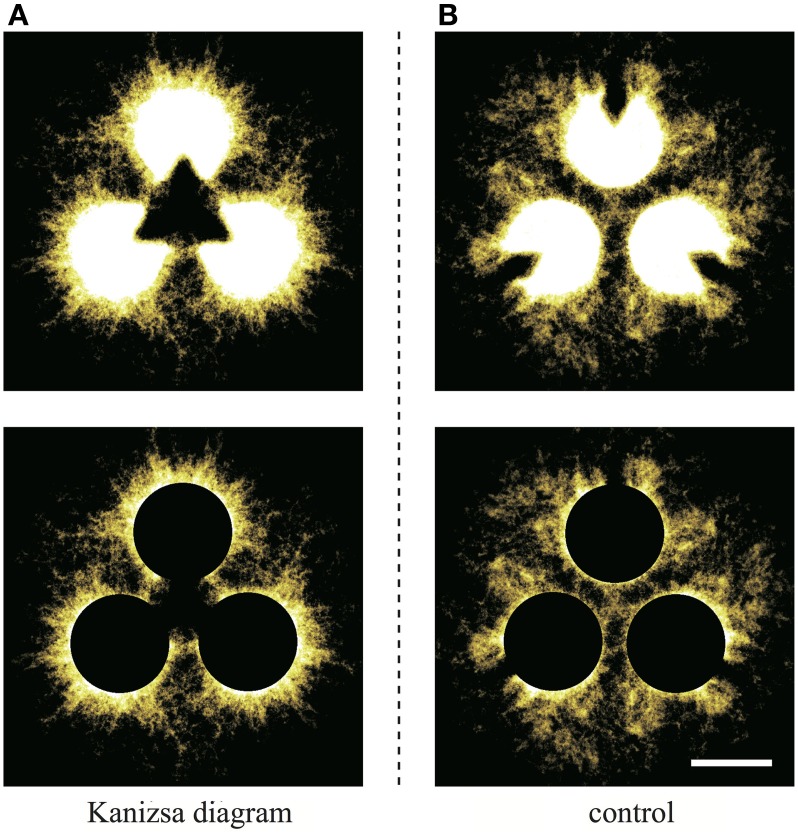
**The upper diagrams are produced by stacking binaralized images of the plasmodial networks for all trials. (A)** Kanizsa diagram and **(B)** control. The brightness of each site indicates the intensity of lapping; that is the existence probability of the plasmodium at the site. A filter that cuts-off the weak signals was applied to improve visibility. This threshold is common to both figures. In the lower diagrams, to wipe out the effect of Kanizsa illusory contour, the pac-men are covered by black (scale bar: 10 mm).

To clarify this, we produce Figure 9 by stacking binarized images for all of the trials. Because the initial configuration has rotational symmetry, the images that are 120° and 240° rotations of the original are also used. In Figure 9, the brightness of each site indicates the intensity of lapping, which is the probability of the plasmodium being at the site. The sites that are occupied more frequently have higher brightness levels. Therefore, the whole image represents the distribution of the probabilities. We have used the “Lookup table/Yellow Hot” color chart in ImageJ. To improve the visibility of the figures, a filter was applied to cut off the weak signals. This threshold is determined as 10% of the theoretical maximum of the signal, and this value is common to both conditions. To remove the effects of Kanizsa illusory contour, the pac-men are covered with black in the lower row. Obviously, the type I-like structure is apparent in the Kanizsa diagram case (Figure 9A), and the structure that is the sum of type I and type IV networks appears in the control (Figure 9B). This observation does not conflict with our model results because type I networks appeared dominantly in the Kanizsa diagram case, but in the control, type I and type IV networks were obtained in almost the same ratio. Next, we emphasize this difference numerically.

**Figure 10:**
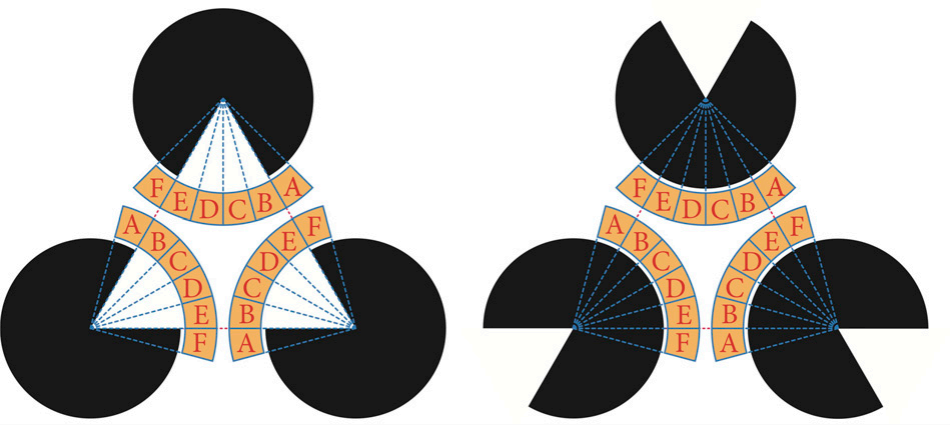
**Target region of analysis**. The marginal areas of the fans that are colored yellow are divided into six regions and named A–F according to the direction. For each divided region, we counted the number of black pixels and estimated the area covered by the plasmodium. An occupation ratio of a region is given as the ratio of covered area to the total area of the region.

**Figure 11:**
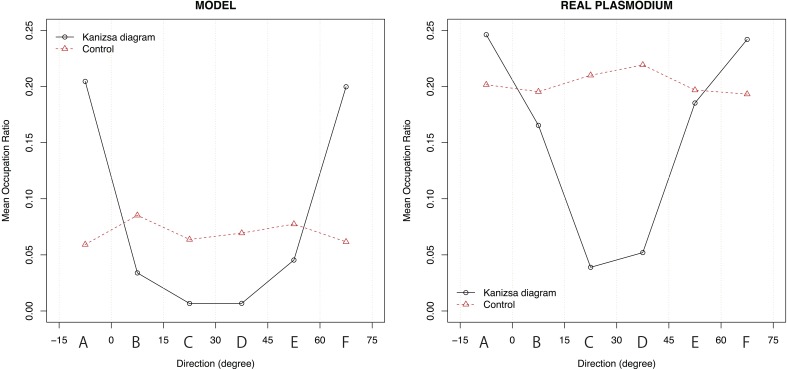
**The relationship between the mean value of the occupation ratio and direction**. For the Kanizsa diagram, there are two peaks at region A and F in both experiments. In the model case, type I and type III networks canceled each other out. Therefore, a clear peak does not appear. There is also no peak in the real plasmodium case because of the isotropic growth of the plasmodium.

We defined the target region of evaluation as depicted in Figure 10. The yellow-colored marginal area of the fan that centers a pac-man was divided into six regions. All of these fans had the same central angle of 15° and were labeled A to F according to the direction from the center. The same labeled areas were identified. For each region, the ratio of the area occupied by plasmodium was measured. We precisely counted the number of black pixels, which indicated that the site was occupied by plasmodia in the binary converted images (see, for example, Figure 5D). To normalize the values, the number of black pixels was divided by the total number of pixels for each region. The relationships between the mean values of the occupation ratios and directions are plotted in Figure 11.

In the Kanizsa diagram cases, the plasmodia converged to areas A and F. In contrast, in the control cases, the *Physarum* spread uniformly, and there was no clear peak. In the model case, there were significant differences in the occupation ratios between the two figures for all areas at a 0.1% level according to the Wilcoxon rank sum test (A: *W* = 74,996, *p* < 0.001; B: *W* = 34,774, *p* < 0.001; C: *W* = 26,542, *p* < 0.001; D: *W* = 26,952, *p* < 0.001; E: *W* = 37,559, *p* < 0.001; F: *W* = 74,480, *p* < 0.001). Similarly, in the case of actual plasmodia, we could see that there were significant differences at the same level for only areas C and D, which occupied the center of fan (A: *W* = 2795.5, *p* = 0.416; B: *W* = 2402, *p* = 0.448; C: *W* = 937, *p* < 0.001; D: *W* = 1085, *p* < 0.001; E: *W* = 2335, *p* = 0.304; F: *W* = 2753, *p* = 0.52). In this regard, the results of the model and the real plasmodia are highly consistent. Namely, the illusory contour of the Kanizsa diagram is complemented by the plasmodia in both cases. These results are derived from the directed cell motility depending on the configuration of the attractant.

## 4. Discussion

These results demonstrate that our visual perception of the illusory contours of the Kanizsa diagram can be emulated by both the asynchronous automata-fashioned model and by real plasmodia as an analogy at the phenomenological level. This finding does not mean that the plasmodia have human-like intelligence or perceptive faculties. It is clear that *Physarum* plasmodia cannot be a direct model of our brain because plasmodia do not have even primitive nervous systems. Nevertheless, we can say that there is abstract homogeneity between plasmodial behavior and nervous activity in our brain. This analogy has meaning at a mechanistic level.

It is known that our consciousness contains perception and sensitivity arising from neural information that is processed in our brains. The causality between particular activities of neurons and our consciousness has not been sufficiently investigated. Nevertheless, we can regard this nervous activity as a type of “computation” that is calculated by complex neuronal firing patterns. In other words, we can say that our consciousness is a product of a program consisting of multiple neural networks. Obviously, we cannot consider that the program is the same as an ordinary computer program at the syntactical level. However, the semantics of the program can be discussed. Taking this perspective, it is possible that the perception arising from brain activity is analogous to plasmodial behavior, which is of a different material and complexity altogether.

To perceive the illusory contours of the Kanizsa diagram, both the differentiation and integration processes are necessary. Depending on the local gradient of the stimuli, detection of the edge of the pac-man corresponds to a differential process, and the combination of the local data based on their global configuration corresponds to an integral one. Therefore, the former is a lower-order process that finds the difference in stimuli in a relatively narrow area on the retina, and the latter is a higher-order process that integrates this information and constructs a visual field. This means that the illusory contours appear with a sort of information contraction from lower to higher orders.

What plasmodial behavior corresponds to the each of differential and integral processes? Plasmodia search their surrounding environment and react to the attractant and the repellant. This is differential. For integration, plasmodia propagate local information using protoplasmic flow to decide their overall behavior. In our model, the imbalance of the choice of SP depending on the local state and the movement of VP based on asynchronous application of the transition rules corresponds to the differential and integral processes, respectively. Therefore, the model and real plasmodia have analogous functionality to the processes of visual perception of the illusory contours. In this way, plasmodia can combine lower and higher-order information smoothly without a specialized pivotal organ, such as a nerve center, that oversees the entire system.

It is important to note that the resulting networks' complementary illusory contour cannot be obtained as an optimized network at the global level. Actually, we can link all of the pac-men with more shorter paths. For example, the V-shaped network (type II) and the Steiner tree (star shape, type IV) have more total shorter paths than the triangular network (type I). However, these networks were not generally obtained in either experiment. If the plasmodia oversee the whole and choose optimized networks, triangular networks would not appear. This means that the illusory contours seem to result from the redundancy of the network, which is considered to be an error or mistake in the *Physarum* computer studies that consider some optimization problems.

In the Kanizsa diagram, the mouth of a pac-man works as an obstacle to change shorter paths and provides metastable networks such as in type I because all pac-men are face-to-face. However, in the control, all pac-men are back-to-back, and the mouths of the pac-men do not behave as barriers. Then, the transition of the network structure to a more stable and shorter network occurs quickly. This indicates that the resulting networks reflect the potential structure of each spatial configuration of pac-men.

The use of an analogy to recognize that plasmodial behavior and visual perception have the same functionality provides us with one perspective about the origin of illusory contours. When we pick out information from our visual field, it is important to determine the borders of the objects using gradients of stimuli such as brightness or color. However, it is inefficient to process all local information at the same level because the volume of information is too huge. Therefore, to efficiently find the border, we must compress the local information to some extent. In this way, we can say that illusory contours are a by-product of the information contraction corresponding to the moderate optimization of the plasmodial networks in our analogy.

We have equated the plasmodial behavior and our perception at the mechanistic level and discussed the analogous homogeneity between them. Finally, we refer to a point of difference between our study and previous studies concerning *Physarum* computation. Whereas we used the true slime mold plasmodium as a computational device for a visual perception model, in conventional works, plasmodia have been used for optimization problems. We can say that the latter viewpoint considers *Physarum* computation to be an effective bio-inspired algorithm, similar to a genetic algorithm or a neural network.

Our perspective is completely different. We are attempting to understand the computational processes of organisms through a model based on bio-computation. A bio-computer is the most suitable device for modeling the computational processes of an organism. To understand biological computational processes, it is necessary and important to construct models of these processes using a bio-computer and to discover the abstract connections between the model and the phenomena. Our experiments are a starting point for *Physarum* computational studies that suggest the computability of our “perception” and “sensitivity.”

## Funding

This work was supported by JSPS KAKENHI Grant Number 25280091.

### Conflict of interest statement

The authors declare that the research was conducted in the absence of any commercial or financial relationships that could be construed as a potential conflict of interest. The Review Editor declares that despite being affiliated to the same institution as the author Yukio-Pegio Gunji, the review process was handled objectively and no conflict of interest exists.
